# Combined external fixation frames and autologous bone graft reconstruction in thumb proximal phalanx bone loss: A case series^✰^

**DOI:** 10.1016/j.jpra.2026.01.019

**Published:** 2026-01-29

**Authors:** Sean Curran, Ciaran Hurley, Safwat Ibrahim, Jamal El-Deib

**Affiliations:** a Department of Plastic and Reconstructive Surgery, Beaumont Hospital, Dublin, Ireland; b Royal College of Surgeons in Ireland, St Stephen’s Green, Dublin, Ireland; c Prince Sultan Military Medical City, Riyadh, Saudi Arabia

**Keywords:** Phalanx, Fracture, Osteomyelitis, Bone loss, Thumb

## Abstract

**Background:**

Proximal phalangeal fractures with significant bone loss are inherently unstable, often resulting in significant malalignment secondary to the high degree of instability. Often significant bone loss is not amenable to surgical fixation alone and bone grafts are required to maintain length and reconstruct the given defect.

**Objective:**

This cases series exhibits three cases of fixation of proximal phalanx fractures of the thumb with associated bone loss using an independently constructed external fixation frame in combination with autologous iliac crest bone graft to restore bone stock and digit length.

**Methods:**

3 patients were included, all of whom suffered proximal phalanx fracture(s) of the thumb with associated bone loss requiring autologous bone graft and external fixation.

**Results:**

All 3 patients achieved radiographic union. The minimum Kapandi opposition score noted at 1 year postoperatively was 8. No postoperative complications were recorded.

**Conclusion:**

We believe that our combined approach of iliac crest bone grafting and independently constructed external fixation frame achieves restoration of bone and digit length with simultaneous fracture reduction and stabilization in cases with significant bony destruction.

## Introduction

The following case series describes comminuted proximal phalangeal fractures of the thumb with associated bone loss, each with a different mechanism of injury.

Comminuted fractures, such as those presented in this case series, are inherently unstable due to their mechanism of injury and fracture pattern, typically requiring surgical fixation.^1^^,^^2^ The techniques employed in surgical fixation are generally a case-specific decision, ranging from basic axial Kirschner wire fixation to open reduction and internal fixation, with a wide variety of techniques described.^3^, ^4^, ^5^

Each of the cases’ management included comprised of surgical fixation with an independently designed external fixation device in combination with autologous bone grafting to reconstruct the area of bone loss. A similar stepwise approach is described throughout each of the following three cases with the resultant external fixation device tailored to the specific fracture/injury.

## Patients and methods

All patients suffered a fracture of the proximal phalanx of the thumb and were treated in either St. James Connolly Hospital, Blanchardstown or Prince Sultan Military Medical Hospital, Riyadh, Saudi Arabia between March 2017 and July 2023. Each patient included had demonstrable bone loss of >/= 5 mm reduction measured on bilateral hand radiograph. The mechanism of injury included a gunshot wound, fracture non-union in the setting of osteomyelitis and osteomyelitis secondary to poorly controlled diabetes, respectively.

Each patient included underwent a minimum of 1 year postoperative follow up. Final clinical radiographs were taken 1 year postoperatively in each case with concomitant Kapandji score assessment.

### Surgical technique

Each external fixation frame was constructed from 2 individual K-wires passed horizontally in the anteroposterior plane through the base of the proximal phalanx and either the distal aspect of the proximal phalanx or the base of the distal phalanx; in cases whereby there is loss or considerable comminution of the distal aspect of the proximal phalanx at the condylar heads. The extra-anatomical component of each wire is then manipulated so that the wires interlock and can be used to maintain the proximal phalanx to length while simultaneously ensuring fracture reduction and stability.

In addition to external fixation each patient underwent autologous iliac crest corticocancellous bone grafting to reconstruct the area of bone loss of the proximal phalanx. The bone was harvested via a curvilinear incision overlying the anterior superior iliac spine, with the harvest tailored to the corresponding proximal phalanx defect.

Case by case variation in management included implantation of antibiotic-impregnated beads in a case of initial Kirschner wire repair of a proximal phalanx fracture with subsequent infection and resultant bone loss secondary to osteomyelitis.

## Case series

### Case 1

**Figure 1:**
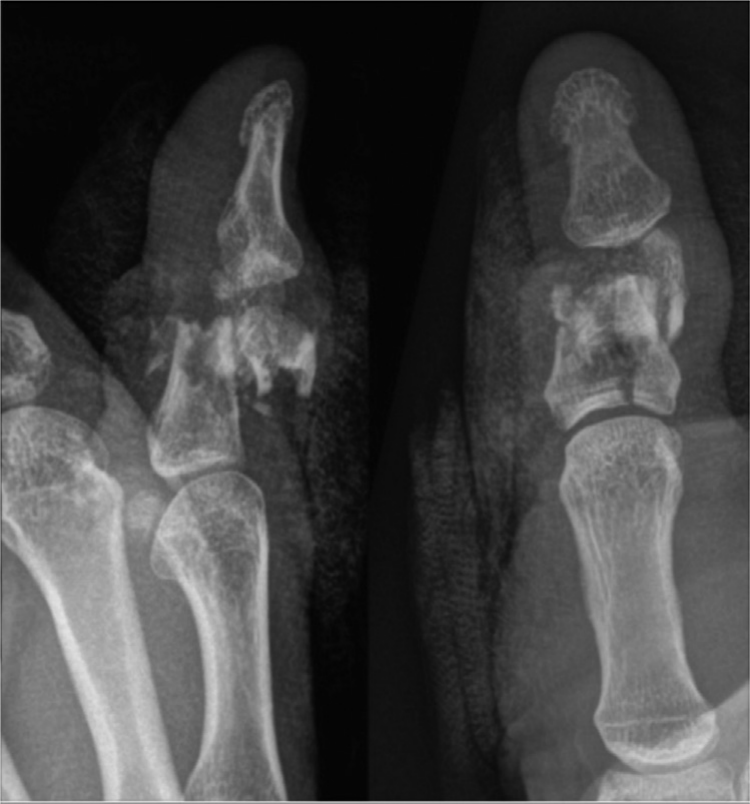
Plain film x-ray demonstrated a comminuted intraarticular fracture of the head and base of the proximal phalanx.

**Figure 2:**
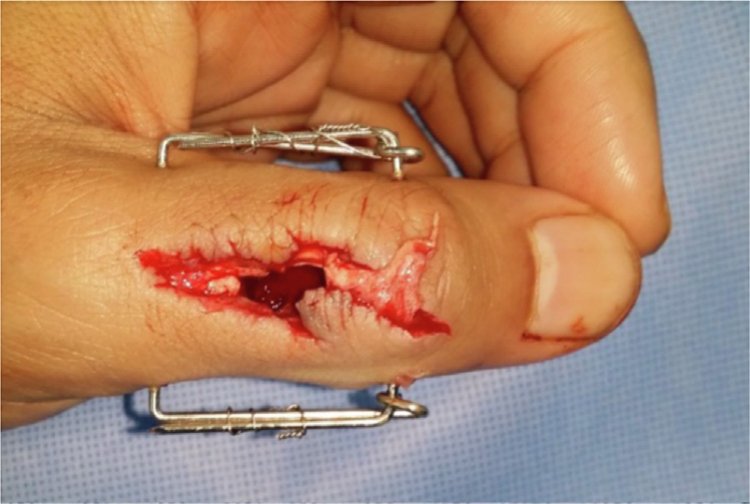
Fracture site debridement demonstrating bone loss; external fixation in-situ.

A 45 year-old male presented with an accidental gunshot injury to his left thumb (Figure 1). He sustained a through and through blast injury. Figure 2 demonstrates this patient with the external fixation device in situ, post-debridement.

### Case 2

**Figure 3:**
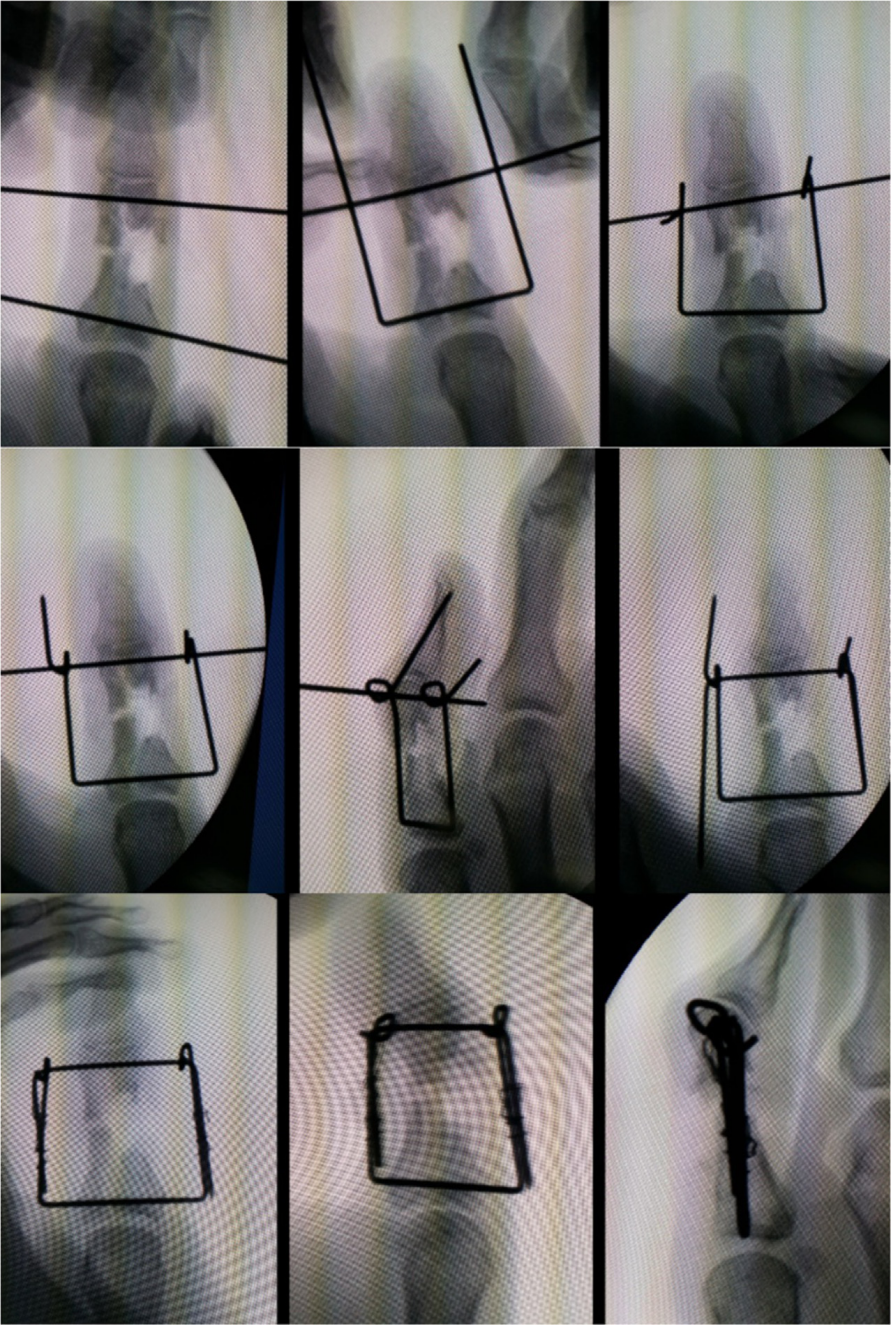
Frame construction under radiological guidance.

**Figure 4:**
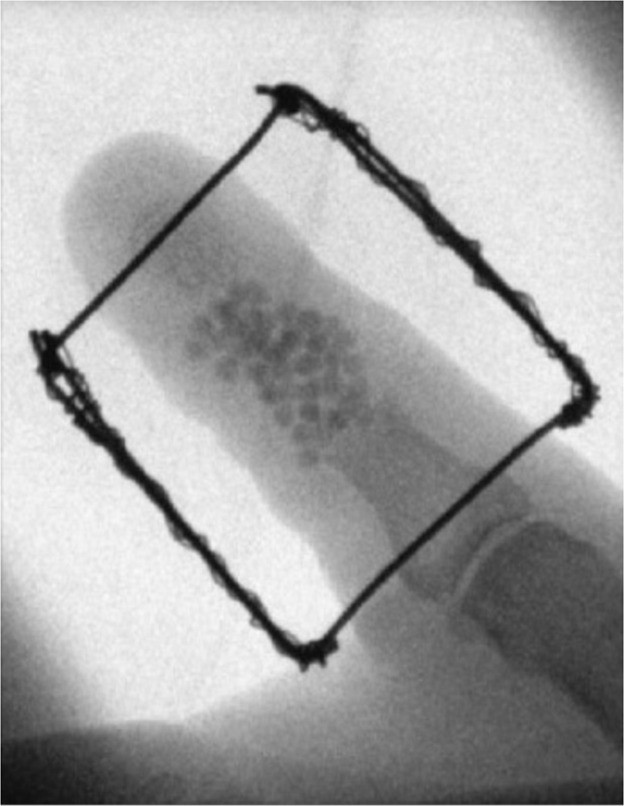
Bony debridement demonstrating bone gap, external fixator and antibiotic beads.

**Figure 5:**
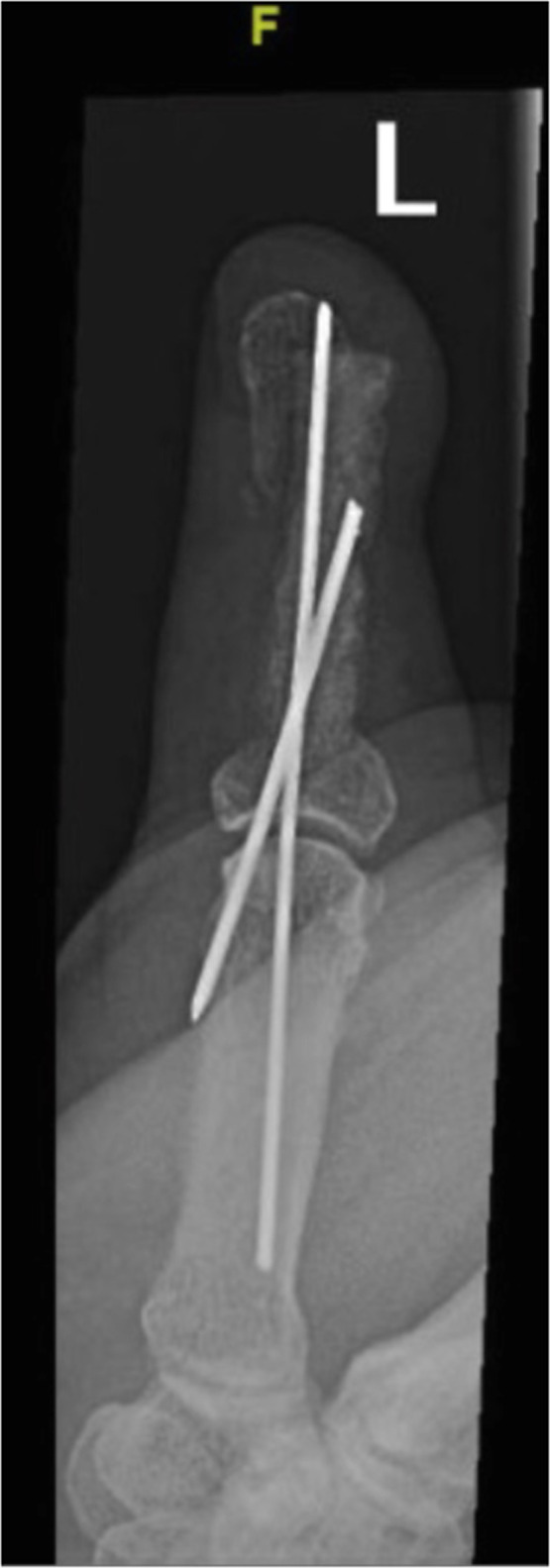
Thumb post iliac bone grafting.

A 62- year old male presented with an open fracture of his proximal phalanx of his right thumb secondary to a high speed fall from an electric scooter which was initially treated with two cross K-wires . Unfortunately, at 5 weeks he developed osteomyelitis, septic arthritis, and fracture non-union as seen clinically and on x-ray. The wires were removed and the bone was extensively debrided, antibiotic impregnated beads were then inserted. An external fixator device was constructed in the same stepwise approach as demonstrated in Figure 3. Figure 4 demonstrates antibiotic-impregnated beads placed at the area of osteomyelitic bone loss. Figure 5 is a radiographic image of the delayed bone graft held in situ with kirschner wires followung eradication of bony infection', after 'An external fixator device was constructed in the same stepwise approach as demonstrated in Figure 3'.

At 6 weeks post antibiotic bead insertion, the second stage reconstruction was performed (Figure 9). The antibiotic beads were removed, and iliac bone graft was harvested and inset with K-wire frame. At 1 year post-operative, the patient was pain free, and demonstrates a Kapandji score of 8 (Video 1).

### Case 3

A 48 year old male with uncontrolled type 2 diabetes mellitus presented with a painful swollen thumb. Plain film x-rays demonstrated severe extensive osteomyelitis of the proximal and distal phalanx. Extensive bony debridement was performed, an external fixator was constructed, and antibiotic beads were inserted. At 6 weeks post-operative, the antibiotic beads were removed, and iliac bone graft was placed. At 1 year, the patient remains pain free, with a functional thumb, and a Kapandji score of 8 (Video 2).

## Discussion

The fundamental principles of open thumb fractures and thumb osteomyelitis were applied throughout each of the above three cases, respectively.^6^^,^^7^ Thereafter, the specific management varied based on each individual case, however the external fixation device was constructed and applied in all three cases in the same stepwise fashion as detailed above.

In each of the respective three cases, delayed bone graft reconstruction was required to reinstate bone length and integrity, with various techniques and application for bone grafts in the setting of significant bone loss in thumb injuries and infection.^8^^,^^9^

While simplistic in design, the described external fixation technique allows tailored treatment of a given fracture, facilitating wire placement at the desired site(s) along with subsequent manipulation and adjustment under direct vision and/or radiographic guidance. Such use of the frame may be applied to injuries of various etiology, as attested to by the cases discussed, and is not limited to a certain length or span across fracture site(s) as may be encountered in the case of commercially pre-constructed frames of a predetermined size.^10^ Additionally, this technique may also be applicable in centers with fewer resources and potentially less access to more commercially available devices.

We believe that this case series demonstrates the benefit of combined autologous bone graft and tailored external fixation fracture repair in cases of severe bony injury/destruction not amenable to surgical fixation alone. This technique allows restoration of bone length while maintaining simultaneous fracture reduction and stability. All three patients commenced hand therapy by 1–2 weeks, in keeping with the rationale of external fixation devices over internal fixation devices in similar injuries.^10^

All patients achieved radiographic union with restoration of thumb length and a minimum Kapandji score of 8 at 1 year postoperatively, with mild limitation in motion deemed acceptable given the severity of initial injury. In summary, the use of iliac crest bone graft combined with external fixation resulted in reliable union, restoration of length, and functional recovery in challenging proximal thumb phalanx fractures. Thus, it serves as an important option when internal fixation alone is unlikely to succeed due to bone loss or compromised soft tissues.

## Funding

None.

## Ethical approval

Not required.

## Declaration of competing interest

None declared.

## Appendix Supplementary materials

**Video 1.** Outcome of Patient 2 at 1 year post-operative. Image, video 1 dummy alt text

**Video 2.** One year post-operative function of Patient 3. Image, video 2 dummy alt text
